# Combination of Chinese medicinal formulas and chemotherapy for triple-negative breast cancer strengthens body resistance to eliminate pathogenic factors

**DOI:** 10.1097/MD.0000000000032350

**Published:** 2022-12-23

**Authors:** Yiyi Zhang, Jing-Wen Mo, Hai-Zhen Lu, Ling-Ling Han, Chengjiang Liu, Yi Zhou

**Affiliations:** a College of Basic Medicine, Chengdu University of Traditional Chinese Medicine, Chengdu, China; b Department of General Medicine, Affiliated Anqing First People’s Hospital of Anhui Medical University, Hefei, China.

**Keywords:** adjuvant therapy, chemotherapy, Chinese medicine formulas, meta-analysis, strengthening the body resistance to eliminate pathogenic factors, triple negative breast cancer

## Abstract

To evaluate the efficacy and safety of strengthening the body’s resistance to eliminate pathogenic factors in Chinese medicinal formulas combined with chemotherapy (hereafter referred to as combined therapy [CT]) in triple-negative breast cancer.

**Methods:**

By searching the 7 electronic databases, PubMed, EMBASE, Web of Science, Cochrane Library, Chinese Academic Journal, Wanfang Database, and Chinese Science and Technology Journal, from the beginning of the establishment to April 2022 to identify eligible randomized controlled trial studies.

**Results:**

The meta-analysis showed that compared with chemotherapy, CT can effectively improve the objective remission rate (risk ratio [RR]: 1.39; 95% confidence interval [CI]: 1.28, 1.52; *P* < .00001, *I*^2^ = 3%), reduce the recurrence rate (RR: 0.33; 95% CI: 0.14, 0.78; *P* = .01, *I*^2^ = 0%) metastasis rate (RR: 0.48; 95% CI: 0.31, 0.73; *P* = .0006, *I*^2^ = 0%) and the incidence of toxic and side reactions, lower tumor marker levels, regulated T lymphocyte subset changes, and increased average progression-free survival (standardized mean difference: 2.78; 95% CI: 1.41, 4.14; *P* < .0001, *I*^2^ = 97%), and improve the quality of life (RR: 1.55; 95% CI: 1.21, 1.99; *P* = .0005, *I*^2^ = 52%).

**Conclusion:**

This study suggests that CT appears to be an effective and safe treatment approach. Although this conclusion requires further confirmation owing to insufficient quality of the included trials.

## 1. Introduction

Triple-negative breast cancer (TNBC) is a breast cancer without estrogen receptor, progesterone receptor, or human epidermal growth factor receptor-2 expression.^[1]^ Compared with hormone receptors or human epidermal growth factor receptor-2 positive breast cancer, TNBC has an early age of onset, strong invasion, high heterogeneity, easy drug resistance, and poor prognosis.^[2]^ A survey showed that TNBCs account for 24% of newly diagnosed breast cancers, and the incidence increases annually.^[3]^ It was reported that about 2088, 849 cases of TNBC were reported in 2018, making it a common cancer in women.^[4]^ Using currently available therapeutic methods, the mean survival of the disease was 5.4 ~10.2 months, With a 5-year survival rate of 65% in local tumor cases and 11% with tumor spread to distant organs.^[5]^

Due to the lack of therapeutic targets, chemotherapy is the primary therapeutic regimen for TNBC. Despite the higher response rate of early TNBC to chemotherapy, long-term treatment is prone to drug resistance and recurrence often occurs in stage III,^[6]^ making chemoresistance a significant challenge.^[2]^ In addition, the toxicity and side effects of chemotherapy drugs cannot be neglected, especially its immunosuppressive effect, which often aggravates the disease.^[7]^ Thus, there is an urgent need to identify new drugs or methods to treat TNBC. For a long time, in China, a combination of traditional Chinese medicine (TCM) and chemotherapy has been widely used in the treatment of TNBC. However, its clinical efficacy and safety need to be confirmed.

From the perspective of TCM, the pathogenesis is mostly caused by positive deficiencies and excess evil.^[8–12]^ Consistently, a survey indicated that positive deficiency and evil excess syndrome accounted for 76.9% of TNBCs.^[13]^ Therefore, the use of Chinese medicinal formulas (CMFs) to strengthen resistance to pathogenic factors is the basic method for curing this disease. CMFs for strengthening healthy qi and eliminating pathogenic factors are composed of Chinese herbal medicines to strengthen healthy qi and eliminate evil. In the treatment of TNBC, Chinese herbal medicine, which replenishes qi, nourishes the blood, nourishes yin, and warms yang, is usually used to help healthy qi. Traditional Chinese medicine for promoting qi, activating blood circulation, clearing away heat and toxins, resolving phlegm, and dispersing knots is used to eliminate evil Qi.^[8]^ In China, combined therapy (CT) is one of the most commonly used treatments for TNBC.^[14]^ However, its efficacy and safety have not been systematically evaluated. Therefore, our study aimed to estimate the efficacy and safety of CT in the treatment through a meta-analysis. The Preferred Reporting Items for Systematic Review and Meta-analysis checklist, http://links.lww.com/MD/I266 and flow diagram, http://links.lww.com/MD/I267 are available in Supplementary Materials.

## 2. Materials and Methods

### 2.1. Inclusion and exclusion criteria randomized controlled trial

#### 2.1.1. Research object.

Patients with a definite diagnosis of TNBC.

#### 2.1.2. Intervention measures.

Control group was treated with conventional chemotherapy and basic treatment. Based on chemotherapy similar to that of the control group, the treatment group was treated with oral CMFs composed of TCM to strengthen healthy qi and eliminate evil. In this meta-analysis, strengthening body resistance drugs was defined as TCM with the effects of tonifying qi, nourishing blood, nourishing yin, and warming yang, as recorded in the Chinese Pharmacopoeia. The drugs used to eliminate pathogenic factors were TCM, with the functions of tonifying qi and blood circulation, clearing away heat and detoxification, resolving phlegm, and dispersing stagnation. These characteristics should be described in future studies.

#### 2.1.3. Outcome index.

① Objective response rate (ORR), ② the incidence of toxicity and side effects, ③ level of tumor markers, ④ changes in T lymphocyte subset, ⑤ improvement in living quality score, and ⑥ rate of recurrence and metastasis, and average progression-free survival (PFS).

#### 2.1.4. Exclusion criteria.

① Acupuncture, moxibustion, and other traditional therapies; ② reviews, abstracts, letters, conference literature, degree literature, case reports, case series reports, and animal experiments; and ③ similar and repeat studies.

### 2.2. Literature retrieval strategy

Two authors searched 7 databases: PubMed, EMBASE, Web of Science, Cochrane Library, Chinese Academic Journal, Wanfang Database, and Chinese Science and Technology Journal, from the beginning of the establishment to April 2022. There were no language restrictions on publications. For studies with incomplete data, we contacted the authors to obtain relevant information. The search keywords were: “Triple Negative Breast Cancer,” “Triple Negative Breast Neoplasms,” “Breast Neoplasm, Triple-Negative, ” “Medicine, Chinese Traditional,” “Traditional Chinese Medicine,” “Zhong Yi Xue.”

### 2.3. Literature screening and data extraction

Two reviewers (Z.Y.Y. and M.J.W.) independently screened the titles and abstracts of each record based on inclusion criteria. For indistinguishable title/abstract records, the full texts were retrieved for further evaluation. Finally, any disagreement was resolved through discussion between the 2 reviewers or consultation with a third reviewer (L.H.Z.). The following data were extracted: author, year of publication, number of patients, age, intervention measures, reference drugs, course of treatment, outcome indicators, and follow-up time.

The Cochrane Handbook (edition 5.1.0) and Review Manager software (edition 5.4.1) were used to assess the risk of bias in the included studies. The risk of bias included 7 aspects: random sequence generation, allocation concealment, blinding of participants and personnel, blinding of outcome assessment, incomplete outcome data, selective outcome reporting, and other sources of bias. Specific risks of bias were divided into high-, low-, and unclear-risk groups. Finally, all studies on selective outcome reporting and other sources of bias are considered at risk of ambiguity. Risk of bias assessment was conducted by 2 authors, and any differences were resolved through discussion between the 2 authors.

Review Manager software (edition 5.4.1) was used for quantitative synthesis. Dichotomy variables were analyzed using risk ratio (RR) and 95% confidence interval (CI). Continuous variables were assessed using the standardized mean difference (SMD) and 95% CI. Heterogeneity was estimated using Cochran’s *Q* test and the *I*^2^ statistic. When *I*^2^ < 50%, the fixed-effects model was adopted, and when 50%<*I*^2^ < 75%, the random-effects model was applied. *I*^2^ > 75% was considered to indicate high heterogeneity, and the source of heterogeneity was analyzed by establishing subgroups. Statistical significance was set at *P* < .05. A funnel chart, which included more than 10 studies, was used to evaluate the publication bias of the outcomes.

## 3. Result

### 3.1. Research inclusion and exclusion process

**Figure 1.**
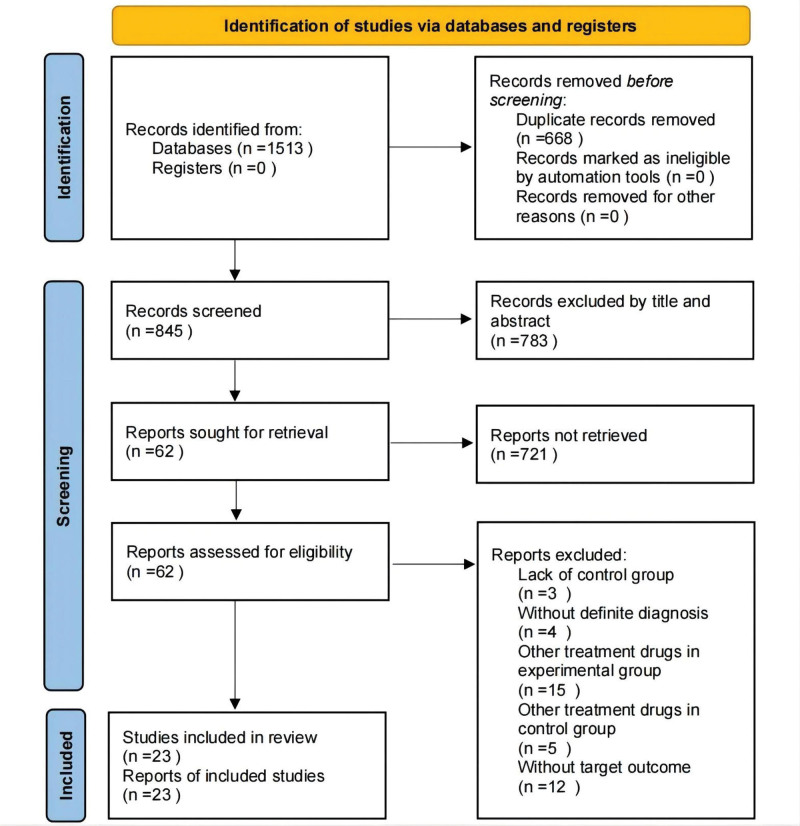
The PRISMA flowchart of the selection process. PRISMA = Preferred Reporting Items for Systematic Review and Meta-analysis.

A total of 1513 studies were identified by searching the databases and 845 articles remained after removing duplicate literature. After layer-by-layer screening, 23 studies were included in this meta-analysis. A flowchart of the selection process is shown in Figure 1.

### 3.2. The basic characteristics of the inclusion study and the results of bias risk assessment

**Figure 2.**
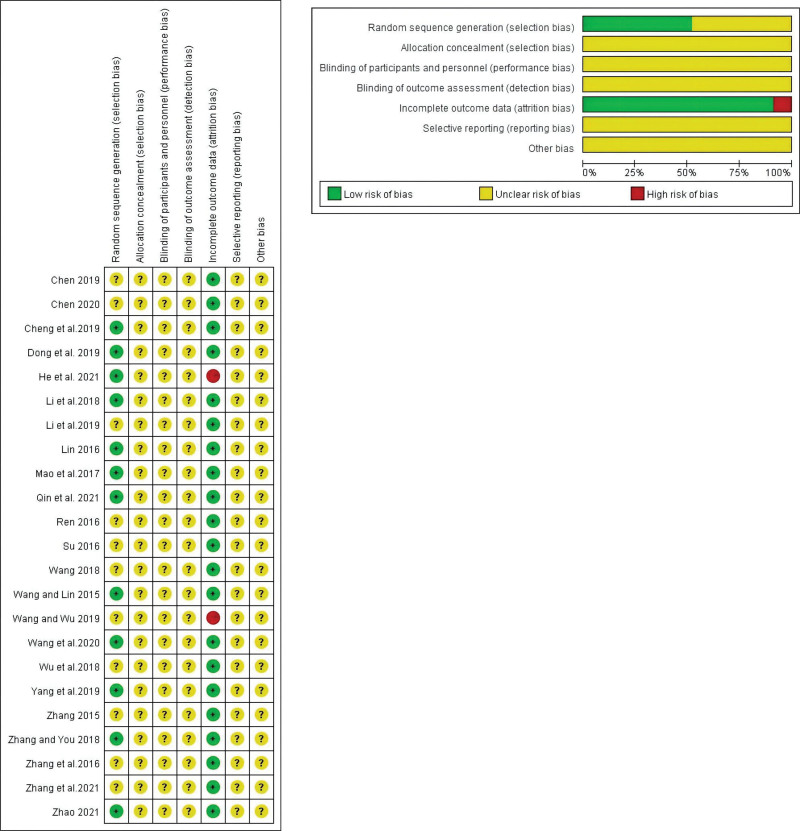
Summary of the risk of bias.

General characteristics of the 23 included studies.^[15–36]^ In 2015 to 2021, 1810 patients were included, including 905 patients in the treatment group and 905 patients in the control group. The general characteristics of the study population are summarized in Table 1. The risk of bias for each study was assessed using the Cochrane risk-of-bias tool. A summary of the risk of bias is shown in Figure 2.

**Table 1:** The general characteristics of the study population.

Study	Sample size	Average age	Fuzheng quxie prescription	Chemotherapy	Course	Outcomes	Follow-up
	Expt./Ctrl.	Expt./Ctrl.					
Chen 2019	40/40	(60.81 ± 3.66)/(61.53 ± 4.37)	Shugan Jianpi Yishen decoction	CTX + PTX	21d*3	(1) (2) (3)	NR
Chen 2020	60/60	(45.20 ± 2.25)/(45.30 ± 2.15)	Guben Ruanjian decoction	GEM + DDP	21d*6	(1) (2) (3) (4)	NR
Cheng et al 2019	33/32	(47.20 ± 2.10)/(49.30 ± 2.50)	Yanghe decoction	GEM + DDP	21d*2	(1) (2) (3) (6)	NR
Dong et al 2019	35/35	(54.75 ± 8.31)/(52.43 ± 7.24)	compound Tubeimu Preparation	GEM/DTX/ECX/DDP	21d*6	(1) (2) (6) (8)	1.5 yr
He et al 2021	28/28	(48.60 ± 5.20)/(51.20 ± 6.50)	Cigu Pingyan decoction	DOX + CTX + DTX	21d*8	(1) (2) (3) (6) (7)	NR
Li et al 2018	30/30	(52.30 ± 10.60)/(51.60 ± 11.10)	Jianpi Bushen decoction	CTX + EOX + PTX	21d*3	(1) (2) (3)	NR
Li et al 2019	31/31	(42.11 ± 12.63)/(41.98 ± 12.59)	Tiaopi Shugan decoction	PTX	21d*6	(1) (2) (3) (4)	NR
Lin 2016	32/33	(49.60 ± 4.30)/(48.50 ± 4.20)	Xingqi Jianpi decoction	CTX + EOX + FU	21d*4	(1) (2) (3) (6) (8)	NR
Mao et al 2017	34/34	(49.50 ± 6.30)/(50.70 ± 3.10)	Fuzheng Hualiu decoction	GEM + DDP	21d*2	(1) (2) (3) (5) (6)	NR
Qin et al 2021	60/60	(57.21 ± 6.90)/(57.03 ± 6.57)	Fuzheng Quxie decoction	DTX/GEM	21d*3	(1) (2) (5) (7)	1 yr
Ren 2016	23/23	(53.90 ± 2.40)/(52.80 ± 2.30)	Shugan Jianpi Yishen decoction	PTX + GEM	21d*5	(1) (2) (3)	NR
Su 2016	30/30	(44.00 ± 2.70)/(46.00 ± 1.30)	Fuzheng mixture	GEM + DDP	21d*3	(1) (2) (3) (5) (6)	NR
Wang 2018	71/71	(53.31 ± 6.85)/(52.94 ± 7.02)	Huangqi Jiedu decoction	PTX + GEM	21d*6	(1) (2) (3) (6) (8)	3 yr
Wang and Lin 2015	39/40	(51.20 ± 2.30)/(50.70 ± 2.10)	Shugan Jianpi Yishen decoction	PTX + GEM	21d*6	(1) (2) (3) (8)	10 mo
Wang and Wu 2019	43/43	(55.12 ± 2.81)/(54.51 ± 3.42)	Huangqi Jiedu decoction	DTX + CTX	21d*4	(1) (2) (6) (7)	NR
Wang et al 2020	32/31	29–68	Shugan Jianpi decoction	GEM + DDP	21d*6	(1) (2) (3) (6)	NR
Wu et al 2018	31/31	(45.24 ± 13.68)/(44.30 ± 12.84)	Shugan Jianpi Yishen decoction	DTX + EOX	15d*4	(1) (2) (3) (4) (5) (8)	2 yr
Yang et al 2019	51/51	(52.43 ± 6.12)/(51.70 ± 6.53)	Huangqi Jiedu decoction	GEM + PTX	21d*6	(1) (2) (3) (5)	NR
Zhang 2015	25/25	(52.31 ± 5.30)/(52.12 ± 4.25)	Shugan Jianpi Yishen decoction	CTX + PTX	21d*5	(1) (2) (3)	NR
Zhang and You 2018	25/25	(43.10 ± 3.70)/(44.60 ± 3.10)	Tiaopi Shugan Yishen decoction	CTX + EOX + ECX	21d*8	(1) (2) (3) (4)	NR
Zhang et al 2016	41/41	(52.00 ± 6.21)/(50.00 ± 7.31)	Fuzheng Xiaoai No. 1 decoction	ALIMTA + DDP	21d*4	(1) (2) (3)	NR
Zhang et al 2021	60/60	(51.04 ± 5.85)/(50.58 ± 5.76)	Kangai Fuzheng decoction	GEM + DDP	28d*6	(1) (2) (3) (5) (8)	NR
Zhao 2021	51/51	(44.54 ± 3.17)/(44.85 ± 4.72)	Shugan Jianpi Jiangni decoction	DTX + EOX + CTX	21d*6	(1) (2) (3) (4) (5) (7)	1 yr

ALIMTA = pemetrexed disodium, Ctrl. = control group, CTX = cyclophosphamide, DDP = cis-platinum, DOX = doxorubicin, DTX = docetaxel, ECX = xeloda, EOX = epirubicin, Expt. = experimental group, FU = fluorouracil, GEM = gemcitabine, PTX = paclitaxel. (1) ORR = objective response rate, (2) DCR = disease control rate, (3) toxic and adverse effects, (4) serum T lymphocyte subsets, (5) serum tumor markers, (6) living quality, (7) recurrence and metastasis rate, (8) average PFS.

### 3.3. Meta-analysis result

#### 3.3.1. ORR.

**Figure 3.**
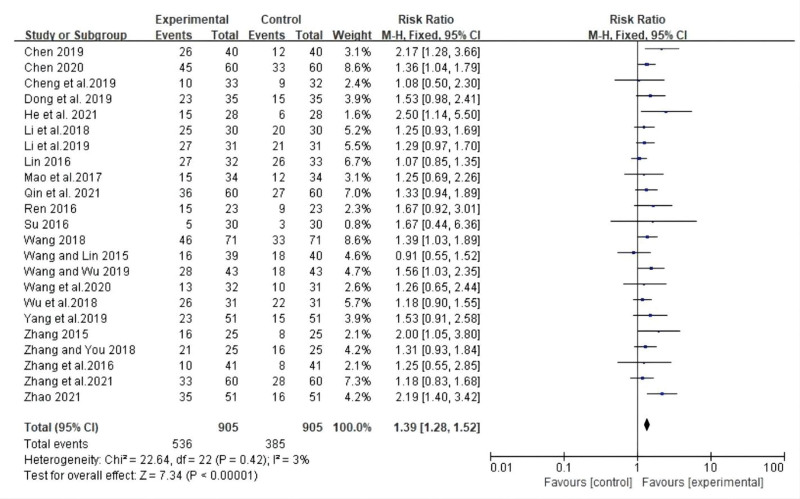
Forest plot for ORR between the experimental and control groups. ORR = objective remission rate.

The ORR is a common indicator for evaluating tumor curative efficacy. Twenty-three studies reported ORR. The ORR in the CT group was higher than that in the chemotherapy group (RR: 1.39; 95% CI: 1.28, 1.52; *P* < .00001, *I*^2^ = 3%). The heterogeneity between the studies was low, and the difference between the 2 groups was statistically significant. A forest plot of ORR is shown in Figure 3.

#### 3.3.2. Recurrence rate and metastasis rate.

**Figure 4.**
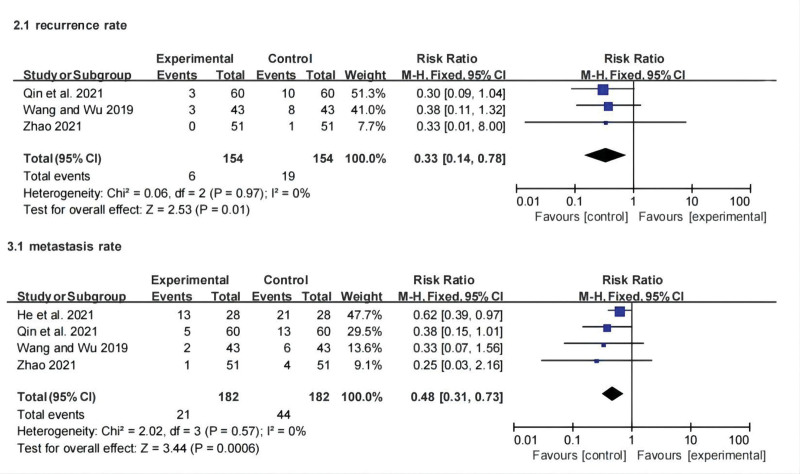
Forest plot for recurrence rate and metastasis rate between the experimental and control groups.

Among the 23 included studies, 3 reported the recurrence rate, and the summary analysis implied that the recurrence rate of the CT group was lower than that of the control group (RR: 0.33; 95% CI: 0.14, 0.78; *P* = .01, *I*^2^ = 0%). Four studies reported the tumor metastasis rate. Similarly, the metastasis rate of CT was lower than that of chemotherapy (RR: 0.48; 95% CI: 0.31, 0.73; *P* = .0006, *I*^2^ = 0%). There was no heterogeneity among the studies, and the combined data showed that the difference between the 2 groups was statistically significant. Forest plots of metastasis and recurrence rates are shown in Figure 4.

#### 3.3.3. Average PFS.

**Figure 5.**
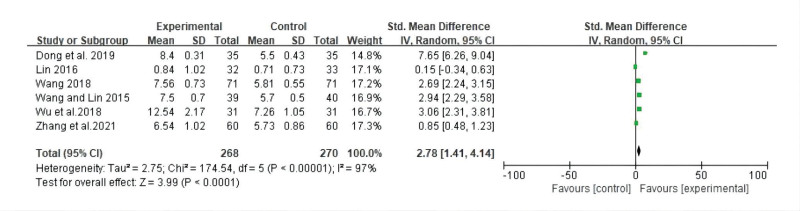
Forest plot for average PFS between the experimental and control groups. PFS = progression-free survival.

Six studies reported average PFS. Compared with the control group, a statistically significant increase in average PFS was observed in the CT group (SMD: 2.78; 95% CI: 1.41, 4.14; *P* < .0001, *I*^2^ = 97%). This result implies that additional TCM treatment to strengthen resistance and eliminate pathogenic factors may be beneficial for increasing the average PFS of patients with TNBC. However, a high degree of heterogeneity was observed among these studies. Moreover, after subgroup analysis according to disease stage or course, heterogeneity was still high. As no source of heterogeneity was found, this result should be treated with caution. A forest plot of the average PFS is shown in Figure 5.

#### 3.3.4. Karnofsky Performance Scale improvement.

**Figure 6.**
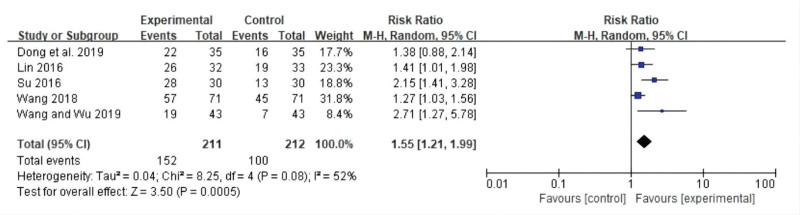
Forest plot for KPS improvement between the experimental and control groups. KPS = Karnofsky Performance Scale.

Five studies reported improvements in Karnofsky Performance Scale (KPS) scores. The meta-analysis indicated that the KPS score improved more in the CT group than in the control group, with a statistically significant difference between the 2 groups (RR: 1.55; 95% CI: 1.21, 1.99; *P* = .0005, *I*^2^ = 52%). Moderate heterogeneity was observed among studies. A forest plot of KPS improvement is shown in Figure 6.

#### 3.3.5. Tumor marker level (Level of CEA, CA15-3 and CA125).

**Figure 7.**
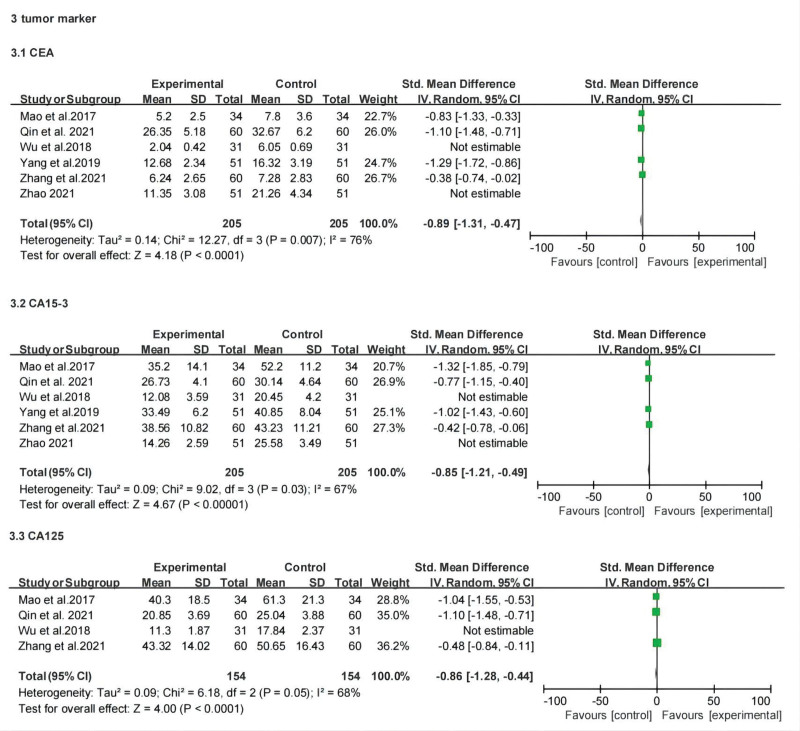
Forest plot for tumor maker level between the experimental and control groups.

Six studies reported levels of carcinoembryonic antigen (CEA) and cancer antigen 15-3 (CA15-3), and 4 studies reported the levels of cancer antigen 125 (CA125). The results indicated that the treatment group of CEA (SMD: −1.99; 95% CI: −2.95, −1.03; *P* < .0001, *I*^2^ = 96%), CA15-3 (SMD: −1.52; 95% CI: −2.31, −0.73; *P* = .0002, *I*^2^ = 94%), and CA125 (SMD: −1.36; 95% CI: −2.18, −0.54; *P* < .001, *I*^2^ = 92%) were all lower than those in the control group, and there were significant differences between the 2 groups. There was high heterogeneity among studies; after excluding studies of Wu et al (2018) and Zhao (2021), the heterogeneity of CEA (SMD: −0.89; 95% CI: −1.31, −0.47; *P* < .0001, *I*^2^ = 76%) and CA15-3 (SMD: −0.85; 95% CI: −1.21, −0.49; *P* < .00001, *I*^2^ = 67%) levels decreased, which implied that they may be the sources of heterogeneity. Similarly, the heterogeneity of CA125 (SMD: −0.86; 95% CI: −1.28, −0.44; *P* < .0001, *I*^2^ = 68%) decreased after excluding the Zhao 2021 study. Our results suggest that CT can effectively reduce tumor marker levels. Forest plots of tumor marker levels are summarized in Figure 7.

#### 3.3.6. Changes of T lymphocyte subsets (serum CD3^+^, CD4^+^, CD8^+^ and CD4^+^/CD8^+^ ratio).

**Figure 8.**
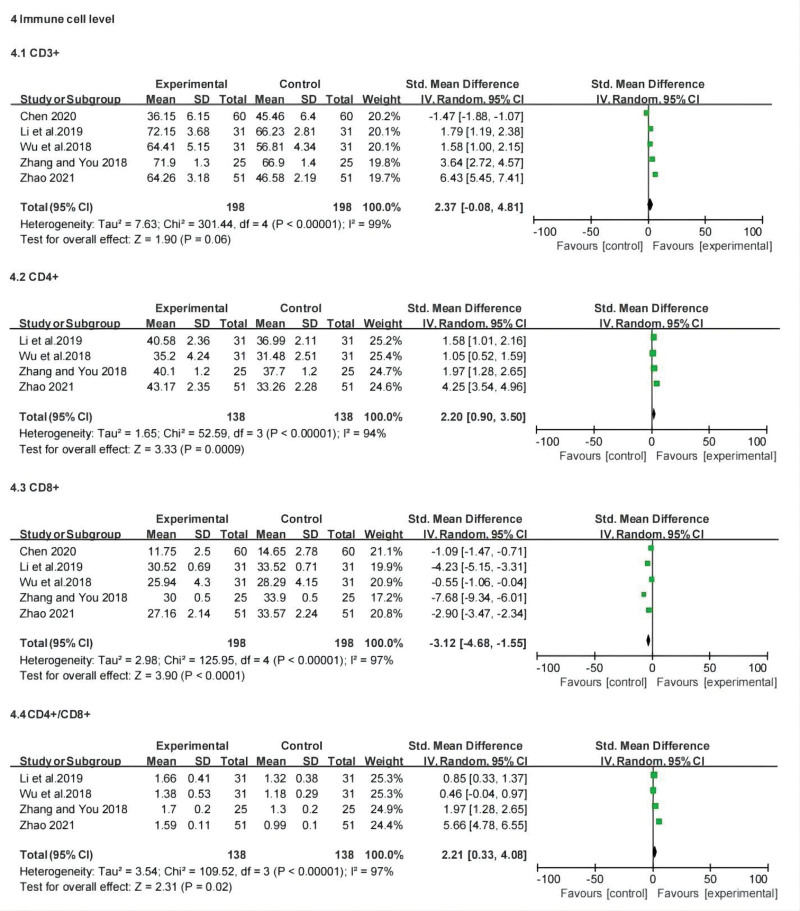
Forest plot for changes of T lymphocyte subsets between the experimental and control groups.

Five studies investigated the percentage of CD3^+^ T cells in the peripheral blood. The meta-analysis showed no significant difference in the percentage of CD3^+^ cells between the treatment and control groups (SMD: 2.37; 95% CI: −0.08, 4.81; *P* = .06, *I*^2^ = 99%). Four studies reported on the percentage of CD4^+^ cells. The percentage of CD4^+^ cells was significantly higher in the treatment group than in the control group (SMD: 2.20; 95% credibility interval: 0.90, 3.50; *P* = .0009, *I*^2^ = 94%). Five studies documented a change in the CD8^+^ percentage. The meta-analysis suggested a lower CD8^+^ percentage in the CT group than in the chemotherapy group (SMD: −3.12; 95% credibility interval: −4.68, −1.55; *P* < .0001, *I*^2^ = 97). Finally, 4 studies reported changes in the CD4^+^/CD8^+^ ratio. The results showed that the ratio of CD4^+^/CD8^+^ cells in the CT group was higher than that in the chemotherapy group (SMD: 2.21; 95% CI: 0.33, 4.08; *P* = .02, *I*^2^ = 97). Overall, the percentage of serum CD4^+^ and the CD4^+^/CD8^+^ ratio was higher than that of the control group, which was lower in the CD8^+^ percentage than in the control group, whereas the percentage of CD3^+^ cells was not significantly different between the 2 groups. Our results imply that additional TCM treatment may reduce the negative impact of chemotherapy on the immune system, thus playing a protective role on the immune system. However, despite data analysis using a random-effects model, SMD model, and subgroup analysis according to disease stage and course of each group, no source of heterogeneity was found. The high heterogeneity among the studies indicates that there are significant differences between them; therefore, we should be cautious about the significance of the results. Forest plots of the changes in T lymphocyte subsets are shown in Figure 8.

#### 3.3.7. Toxic and side effects.

The protective effect of CMFs for strengthening the body and eliminating pathogenic factors is evaluated by the incidence of toxicity and side effects. The main toxic effects reported in the included studies were adverse digestive tract effects, bone marrow suppression, alopecia, leukocyte decline, and platelet reduction.

##### 7.1.3.3. Gastrointestinal adverse reactions.

Seven studies have reported the overall incidence of gastrointestinal adverse effects, 8 studies recorded the incidence of mild to moderate (grade I, II) gastrointestinal adverse reactions, 9 studies recorded the incidence of moderate to severe (grade III, IV) digestive tract adverse reactions, The results of the meta-analysis suggested in the overall incidence of (RR: 0.59; 95% CI: 0.47, 0.73; *P* < .00001, *I*^2^ = 23%) with moderate to severe (grade II, III, IV) digestive tract adverse reactions incidence aspect, the incidence of gastrointestinal adverse reactions in the treatment group was lower than that in the control group, and the difference between the 2 groups was statistically significant. There was no statistically significant difference between the 2 groups in the incidence of mild (grade I) gastrointestinal adverse reactions between the 2 groups.

##### 7.2.3.3. Myelosuppression.

Three studies reported the total incidence of myelosuppression, 6 studies recorded the incidence of mild to moderate (grade I and II) myelosuppression, 6 studies recorded moderate to severe (grade III and IV) myelosuppression. The results showed that in terms of the total incidence (RR: 0.61; 95% CI: 0.43, 0.85; *P* < .00001, *I*^2^ = 6%) and the incidence of moderate and severe (grade III, IV) myelosuppression, the incidence of myelosuppression in the treatment group was lower than that in the control group, and the difference between the 2 groups was statistically significant. However, no significant difference was observed in the incidence of mild-to-moderate myelosuppression (grades I and II) between the 2 groups.

##### 7.3.3.3. Other toxic and side effects.

Finally, 4 studies reported the incidence of alopecia and leukocytosis, and 3 studies documented the incidence of thrombocytopenia. There was no significant difference in the incidence of hair loss or leukopenia between the treatment and control groups. The incidence of thrombocytopenia in the CT group was lower than that in the chemotherapy group and the difference between the 2 groups was statistically significant (RR: 0.56; 95% CI: 0.32, 0.96; *P* = .03, *I*^2^ = 0%).

**Figure 9.**
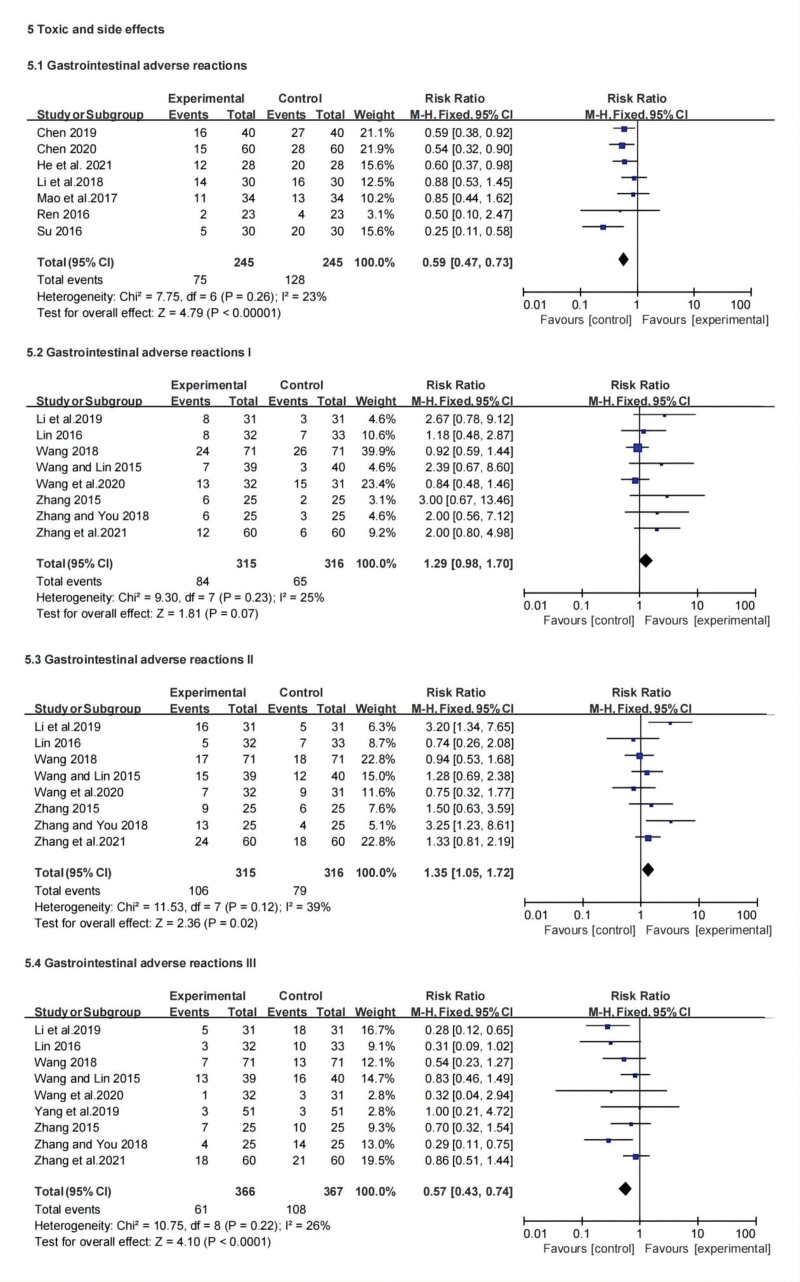
Forest plot for toxic and side effects between the experimental and control groups.

Overall, compared with chemotherapy, CT had lower toxicity and side effects in terms of gastrointestinal adverse reactions, myelosuppression, moderate and severe gastrointestinal adverse reactions, myelosuppression, and thrombocytopenia, whereas there was no significant improvement in the incidence of mild gastrointestinal adverse reactions, myelosuppression, alopecia, and leukopenia. Therefore, TCM may reduce the toxic side effects associated with chemotherapy. Forest plots of toxicity and side effects are shown in Figure 9.

#### 3.3.8. Funnel chart.

**Figure 10.**
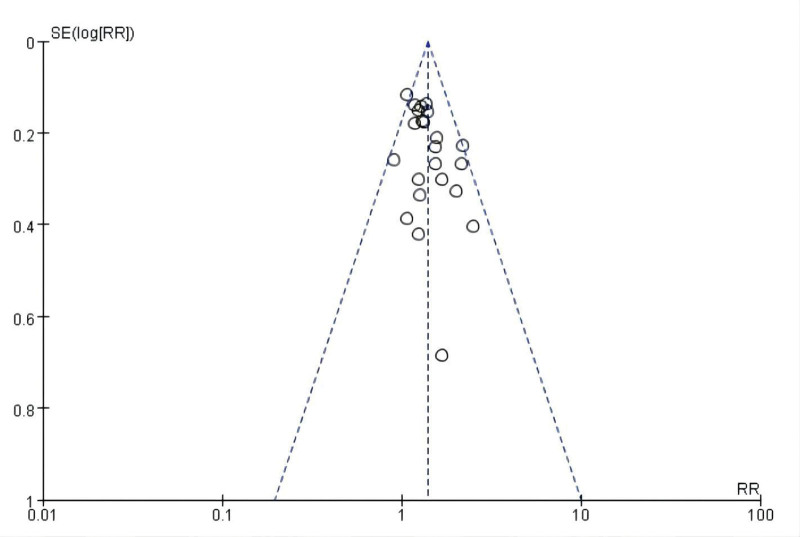
Funnel plots for publication bias of ORR. ORR = objective remission rate.

Publication bias of the ORR was assessed using a funnel plot. The funnel plot of the ORR was not symmetrical, suggesting the existence of publication bias. The remaining studies contained fewer than 10 studies; therefore, publication bias was not evaluated. Funnel plots for the publication bias of the ORR are shown in Figure 10.

## 4. Discussion

Compared with other subtypes of breast cancer, TNBC is characterized by high malignancy, insidious onset, strong invasion, high recurrence and metastasis rates, and short overall survival, making the vast majority of patients with poor prognosis.^[37]^ Chemotherapy is the current treatment, which has drawbacks, such as drug resistance and toxic side effects, necessitating the development of new treatment measures.

In China, TCM has been widely used as adjuvant therapy for chemotherapy in the treatment of TNBC. In addition, epidemiological investigations have shown that “ body resistance weakened while pathogenic factors prevailing” is the most common TCM syndrome in patients with TNBC. Recent studies have revealed possible anti-tumor mechanisms of CMFs for strengthening vital energy and expelling pathogenic factors. Furthermore, researchers have found that drugs that strengthen vital energy and eliminate pathogenic factors may play an anti-tumor role in different ways.^[38]^

### 4.1. Anti-tumor mechanisms of righting drugs

Righting drugs can enhance the healthy qi of the body, prevent invasion by external pathogens, and protect the body. Their anti-tumor efficacy is mainly reflected in 2 aspects: to regulate immune function and then resist the invasion of tumor cells by improving the levels of T and B lymphocytes, and macrophages and regulating the levels of cancer suppression-related cytokines, such as interleukin-2, interleukin-12, and tumor necrosis factor-α.^[39]^ Song et al^[40]^ showed that icariin from Epimedium, a yangtonic drug, can effectively regulate the tumor immunosuppression microenvironment. Its mechanism is related to the downregulation of programmed death-ligand 1 expression, increasing the proportion of infiltrating CD4^+^/CD8^+^ T cells and reducing the content of Myeloid-derived suppressor cells in tumors. To reduce the toxicity and side effects of chemotherapy drugs in the body, organ-protective, and anti-fatigue effects.^[39]^ Chen et al^[41]^ observed the effect of Ganoderma lucidum spore oil on the level of peripheral blood cells in Mouse sarcoma S180 cells tumor-bearing mice after 5 fluorouracil (5-FU) chemotherapy and found that Ganoderma lucidum spore oil can reduce the toxic and side effects of leukopenia and thrombocytopenia caused by 5-FU chemotherapy, suggesting that righting drugs can improve the hematopoietic dysfunction of bone marrow caused by chemotherapy to a certain extent. Additionally, ganoderma acid reduced peripheral muscle fatigue-like behavior induced by 5-FU and improved mitochondrial function of the muscle by suppressing Adenosine monohposphate activated protein kinase, Interleukin-6, and Tumor necrosis factor-α expression in the skeletal muscle, increasing glycogen content and Adenosine Triphosphate production, and reducing lactic acid content and Lactate dehydrogenase activity.^[42]^

### 4.2. Anti-tumor mechanism of anti-pathogenic drugs

The anti-tumor effect of anti-pathogenic drugs is mainly reflected in the effective suppression of tumor cell self-proliferation and angiogenesis, induction of tumor cell apoptosis, and autophagy.^[43]^ A commonly used qi-tonifying drug for TNBC, Szechwan Chinaberry fruit, whose extracts have inhibitory effects on TNBC cells, such as Human breast cancer cells, Breast ductal carcinoma cells, and Mouse breast cancer cells, can induce necrosis, apoptosis, and autophagy.^[44]^

**Figure 11.**
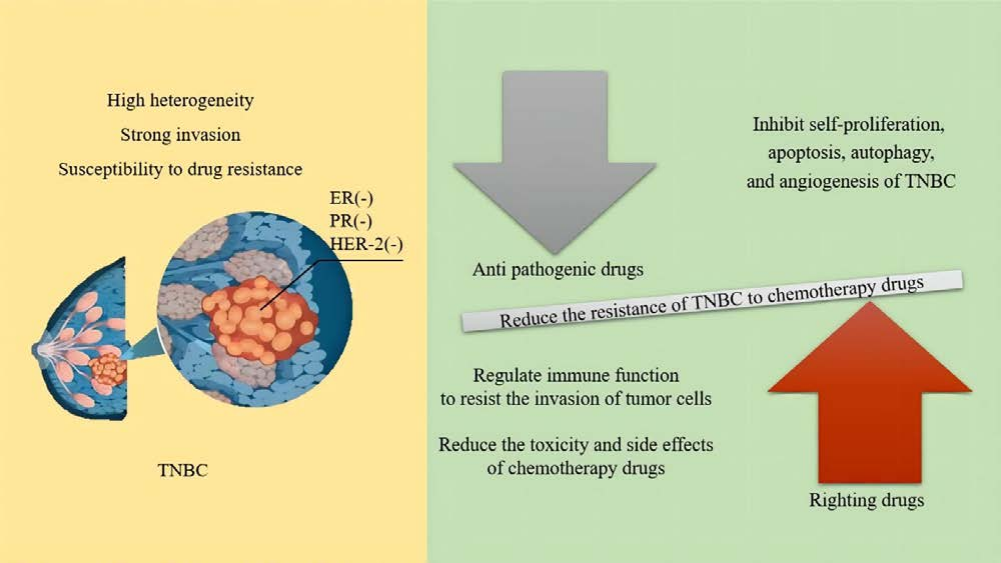
Anti-tumor mechanism of righting and anti-pathogenic drugs.

Moreover, it was found that both righting and anti-pathogenic drugs can reduce the resistance of TNBC to chemotherapy drugs, thus improving the efficacy of chemotherapeutic drugs.^[45]^ In summary, the anti-tumor mechanism of TCM is mostly related to immunity regulation, organ protection, fatigue resistance, tumor inhibition, and sensitivity to chemotherapy drug amplification. Anti-tumor mechanism of righting and anti-pathogenic drugs is presented in Figure 11.

This study was the first to evaluate the efficacy and safety of a combination of the most commonly used TCM formulas and chemotherapeutic drugs for TNBC treatment. First, to evaluate the clinical efficacy of CT, ORR and tumor marker levels, which can reflect tumor progression, were selected as outcomes. These results suggest that TCM can improve the efficacy of chemotherapy drugs by lowering the ORR and the level of tumor markers. Another issue highlighted in our study was the safety of CT. The incidence of toxic effects and changes in T cell subsets were also evaluated. The result indicates that TCM can reduce the incidence of gastrointestinal adverse reactions, bone marrow suppression, and thrombocytopenia. CMFs have a particularly significant effect in reducing the rate of moderate and severe gastrointestinal adverse reactions and bone marrow suppression. Furthermore, since immunosuppression is prevalent during chemotherapy, the percentage of serum T lymphocytes was assessed. The serum CD4^+^ percentage and CD4^+^/CD8^+^ ratio were higher than those in the control group and lower in CD8^+^ percentage than the control group, manifesting that additional TCM may reduce the negative effects of chemotherapy on the immune system. Furthermore, to study the long-term efficacy of CT, PFS, recurrence, and metastasis rates were assessed. In this meta-analysis, CT prolonged the average PFS and lowered the rates of recurrence and metastasis in patients, which may be attributed to sensitivity increase to chemotherapeutic drugs. However, the number of studies was small, making the evidence insufficient, which needs to be verified in future studies. Finally, quality of life was also considered and evaluated by the improvement in KPS scores. Meta-analysis indicated that CT can improve the quality of life of patients with TNBC, which may benefit from the effects of righting drugs. In general, TCM is expected to become a new adjuvant chemotherapy regimen for enhancing the efficacy and reducing the toxicity and side effects of chemotherapy.

This study has some limitations. First, the included studies had poor overall quality. Only a few of the included trials explicitly mentioned the specific method of randomization, and hidden and blinded information was lacking in all studies, which could lead to the generation of bias. However, it should be noted that it is difficult to hide the process of drug allocation in studies using TCM decoctions as interventions. Second, several results showed significant heterogeneity. The source of heterogeneity was not clearly defined despite the use of subgroup analysis and exclusion criteria. This phenomenon may be attributed to the differences between the various prescriptions used in each study. Significant heterogeneity reduced the strength of the evidence, suggesting that the results of the analysis should be interpreted with caution. Finally, because the included studies were conducted in China, it was difficult to evaluate the efficacy and safety of TCM in different ethnic groups and regions. Given these limitations, we recommend conducting large-scale, multicenter, high-quality clinical trials worldwide.

## 5. Conclusion

This study indicated that strengthening body resistance to eliminate pathogenic factors CMFs is a potential adjuvant chemotherapy method for TNBC, which not only improves the ORR, reduces the incidence of toxicity and side effects, recurrence and metastasis rate, and the level of tumor markers, but also enhances cellular immune function, prolongs average PFS, and improves the quality of life. Although these conclusions require further confirmation owing to the existing limitations, the current evidence provides hope for researchers to explore further.

## Author contributions

YZ and JM contributed equally to the study. All the authors contributed substantially to the design, interpretation of the data, statistical analysis, drafting of the manuscript, and approval of submission.

**Data curation:** Yiyi Zhang, Jing-Wen Mo, Ling-Ling Han.

**Formal analysis:** Yiyi Zhang.

**Writing – original draft:** Yiyi Zhang, Jing-Wen Mo.

**Writing – review & editing:** Yiyi Zhang, Hai-Zhen Lu, Yi Zhou, Chengjiang Liu.

## Supplementary Material
