# Supplementary material for: Combination of Chinese medicinal formulas and chemotherapy for triple-negative breast cancer strengthens body resistance to eliminate pathogenic factors
Source: Medicine (Baltimore). 2022 Dec 23;101(51):e32350. doi: 10.1097/MD.0000000000032350 (PMC9794332; doi:10.1097/MD.0000000000032350)
Supplement: Supplementary file 1 [file medi-101-e32350-s001.pdf]

PRISMA 2020 Checklist

Section and Topic	Item #	Checklist item	Location where item is reported
**TITLE**			
Title	1	Combination of Chinese medicinal formulas and chemotherapy for Triple-negative breast cancer strengthens body resistance to eliminate pathogenic factors	Reported on Page No.1
**ABSTRACT**			
Abstract	2	Background: To evaluate the efficacy and safety of strengthening the body's resistance to eliminate pathogenic factors in Chinese medicinal formulas (CMFs) combined with chemotherapy (hereafter referred to as combined therapy [CT]) in Triple-negative breast cancer (TNBC).<br>Method: By searching the 7 electronic databases, PubMed, EMBASE, Web of Science, Cochrane Library, Chinese Academic Journal (CNKI), Wanfang Database, and Chinese Science and Technology Journal (CQVIP), from the beginning of the establishment to April 2022 to identify eligible randomized controlled trial (RCT) studies.<br>Result: The meta-analysis showed that compared with chemotherapy, CT can effectively improve the objective remission rate(ORR)(RR:1.39;95% CI:1.28,1.52;p< 0.00001, I2 = 3%),reduce the recurrence rate(RR:0.33;95%CI:0.14,0.78;p=0.01, I2 = 0%) metastasis rate(RR:0.48;95%CI:0.31,0.73;p=0.0006, I2 = 0%)and the incidence of toxic and side reactions, lower tumor marker levels, regulated T lymphocyte subset changes, and increased average progression-free survival(PFS)(SMD:2.78;95%CI:1.41,4.14; p<0.0001, I2=97%), and improve the quality of life (RR:1.55;95%CI:1.21,1.99;p=0.0005, I2 = 52%).<br>Conclusion: This study suggests that CT appears to be an effective and safe treatment approach. Although this conclusion requires further confirmation owing to insufficient quality of the included trials.	Reported on Page No.1
**INTRODUCTION**			
Rationale	3	Compared with other subtypes of breast cancer, TNBC is characterized by high malignancy, insidious onset, strong invasion, high recurrence and metastasis rates, and short overall survival, making the vast majority of patients with poor prognosis38. Chemotherapy is the current treatment, which has drawbacks, such as drug resistance and toxic side effects, necessitating the development of new treatment measures.<br>For a long time, in China, a combination of traditional Chinese medicine(TCM) and chemotherapy has been widely used in the treatment of TNBC. However, its clinical efficacy and safety need to be confirmed.	Reported on Page No.2
Objectives	4	To evaluate the efficacy and safety of strengthening the body's resistance to eliminate pathogenic factors in Chinese medicinal formulas (CMFs) combined with chemotherapy (hereafter referred to as combined therapy [CT]) in Triple-negative breast cancer (TNBC).	Reported on Page No.1
**METHODS**			
Eligibility criteria	5	Studies in line with the following inclusion criteria were enrolled for this research:<br>1)Participants: Patients with a definite diagnosis of TNBC.<br>2) Invention: Based on chemotherapy similar to that of the control group, the patients in the treatment group were treated with oral CMFs composed of TCM to strengthen healthy qi and eliminate evil. In this meta-analysis, strengthening body resistance drugs was defined as TCM with the effects of tonifying qi, nourishing blood, nourishing yin, and warming yang, as recorded in the Chinese Pharmacopoeia. The drugs used to eliminate pathogenic factors were TCM, with the functions of tonifying qi and blood circulation, clearing away heat and detoxification, resolving phlegm, and dispersing stagnation. These characteristics should be described in future studies.<br>3) Control: Conventional chemotherapy and basic treatment;<br>4)Results: The ORR was the main outcome of this meta-analysis. Secondary outcome measures were the incidence of toxicity and side effects, level of tumor markers, changes in T lymphocyte subset, improvement in living quality score, rate of recurrence and metastasis, and average PFS. Primary outcomes must be reported in the original literature, whereas secondary outcomes require inclusion of at least one item.<br>5) Study type: clinical randomized controlled trial (RCT).<br>Studies that met the following criteria were excluded:	Reported on Page No.4

PRISMA 2020 Checklist

Section and Topic	Item #	Checklist item	Location where item is reported
		1) Invention: acupuncture, moxibustion and other traditional therapies will be excluded;<br>2) Study type: reviews, abstracts, letters, conference literature, degree literature, case reports, case series reports, and animal experiments.<br>3)Similar and repeat studies.	
Information sources	6	Two authors searched seven electronic databases: PubMed, EMBASE, Web of Science, Cochrane Library, Chinese Academic Journal (CNKI), Wanfang Database, and Chinese Science and Technology Journal (CQVIP), from the beginning of the establishment to April 2022.	Reported on Page No.3
Search strategy	7	Two authors searched seven electronic databases: PubMed, EMBASE, Web of Science, Cochrane Library, Chinese Academic Journal (CNKI), Wanfang Database, and Chinese Science and Technology Journal (CQVIP), from the beginning of the establishment to April 2022. There were no language restrictions on publications. For studies with incomplete data, we contacted the authors to obtain relevant information. The search keywords were: “Triple Negative Breast Cancer,” “Triple Negative Breast Neoplasms,” “Breast Neoplasm, Triple-Negative, ” “Medicine, Chinese Traditional,” “Traditional Chinese Medicine,” “Zhong Yi Xue”.	Reported on Page No.3
Selection process	8	Two reviewers independently screened the titles and abstracts of each record based on inclusion criteria. For indistinguishable title/abstract records, the full texts were retrieved for further evaluation. Finally, any disagreement was resolved through discussion between the two reviewers or consultation with a third reviewer.	Reported on Page No.5
Data collection process	9	The included studies were independently reviewed by two authors (ZYY and MJW), and the following data were extracted: author, year of publication, number of patients, age, intervention measures, reference<br>drugs, course of treatment, outcome indicators (objective remission rate, incidence of toxic side effects, level of tumor markers, changes in T lymphocyte subsets, improvement in quality of life score, recurrence and metastasis rate, and progression-free survival), and follow-up time. Any disagreements were resolved through discussion between the two authors.	Reported on Page No.5
Data items	10a	author, year of publication, number of patients, age, intervention measures, reference<br>drugs, course of treatment, outcome indicators (objective remission rate, incidence of toxic side effects, level of tumor markers, changes in T lymphocyte subsets, improvement in quality of life score, recurrence and metastasis rate, and progression-free survival), and follow-up time.	Reported on Page No.5
	10b	course of treatment, follow-up time.	Reported on Page No.5
Study risk of bias assessment	11	The Cochrane Handbook 5.1.0 and Review Manager software (edition 5.4.1)[RevMan5.4.1] were used to assess the risk of bias in the included studies. The risk of bias included seven aspects: random sequence generation, allocation concealment, blinding of participants and personnel, blinding of outcome assessment, incomplete outcome data, selective outcome reporting, and other sources of bias. Specific risks of bias were divided into high-, low-, and unclear-risk groups. Finally, all studies on selective outcome reporting and other sources of bias are considered at risk of ambiguity. Risk of bias assessment was conducted by two authors, and any differences were resolved through discussion between the two authors.	Reported on Page No.6
Effect measures	12	RevMan5.4.1 was used for quantitative synthesis. Dichotomy variables were analyzed using risk ratio (RR) and 95% confidence interval (CI). Continuous variables were assessed using the standardized mean difference (SMD) and 95% CI. Heterogeneity was estimated using Cochran's Q test and the I2 statistic. When I2 < 50%, the fixed-effects model was adopted, and when 50%<I2< 75%, the random-effects model was applied. I2 > 75% was considered to indicate high heterogeneity, and the source of heterogeneity was analyzed by establishing subgroups. Statistical significance was set at P < 0.05. A funnel chart, which included more than 10 studies, was used to evaluate the publication bias of the outcomes.	Reported on Page No.6
Synthesis methods	13a	tabulating the study intervention characteristics and comparing against the planned groups for each synthesis.	Reported on Page No.4
	13b	For indistinguishable title/abstract records, the full texts were retrieved for further evaluation. Finally, any disagreement was resolved through	Reported

Section and Topic	Item #	Checklist item	Location where item is reported
		discussion between the two reviewers or consultation with a third reviewer.	on Page No.5
	13c	General characteristics of the study included are shown in table 1.	Reported on Page No.7
	13d	RevMan5.4.1 was used for quantitative synthesis. Dichotomy variables were analyzed using risk ratio (RR) and 95% confidence interval (CI). Continuous variables were assessed using the standardized mean difference (SMD) and 95% CI. Heterogeneity was estimated using Cochran's Q test and the I2 statistic. When I2 < 50%, the fixed-effects model was adopted, and when 50%<I2< 75%, the random-effects model was applied. I2 > 75% was considered to indicate high heterogeneity.	Reported on Page No.6
	13e	and the source of heterogeneity was analyzed by establishing subgroups.	Reported on Page No.6
	13f	A funnel chart, which included more than 10 studies, was used to evaluate the publication bias of the outcomes.	Reported on Page No.6
Reporting bias assessment	14	In terms of allocation concealment, all studies were unclear because of a lack of detailed information. For the blind method of participants and staff and the blind method of result evaluation, the risks in all studies were unclear owing to a lack of details. For incomplete outcome data, two studies missed the follow-up time for recurrence rate, and the authors were not contacted. Thus, they were judged to be at a high risk.	Reported on Page No.7
Certainty assessment	15	Dichotomy variables were analyzed using risk ratio (RR) and 95% confidence interval (CI). Continuous variables were assessed using the standardized mean difference (SMD) and 95% CI. Statistical significance was set at P < 0.05.	Reported on Page No.8
**RESULTS**			
Study selection	16a	A total of 1,513 studies were identified by searching the databases and 845 articles remained after removing duplicate literature. After reviewing the titles and abstracts, 644 non-RCTs and 139 studies with inconsistent study objectives were excluded and 62 articles were retained. Finally, through a review of the full text, 39 studies that did not meet the criteria were excluded, of which three lacked a control group, four had no clear diagnosis, 15 lacked measures in the treatment group, five lacked measures in the control group, and 12 lacked target outcome indicators. Finally, 23 studies were included in this meta-analysis. A flowchart of the selection process is shown in Figure 1.	Reported on Page No.6
	16b	None.	
Study characteristics	17	General characteristics of the 23 included studies 15-37. In 2015 – 2021,1,810 patients were included, including 905 patients in the treatment group and 905 patients in the control group. All 23 studies used CF as the treatment group measure, and conventional chemotherapy and basic treatment as the control group measures. All the 23 studies were conducted in China. The general characteristics of the study population are summarized in Table 1.	Reported on Page No.7
Risk of bias in studies	18	A summary of the risk of bias is shown in Figure 2.	Reported on Page No.8
Results of individual studies	19	The general characteristics of the study population are summarized in Table 1.	Reported on Page No.7
Results of syntheses	20a	The risk of bias for each study was assessed using the Cochrane risk-of-bias tool. As shown in the figure, the risk of ambiguous bias accounts for the vast majority of cases, because the information provided by many studies is insufficient to judge whether there is a risk of bias. All the trials were RCT and described the inclusion/exclusion criteria and grouping methods. Of the 23 RCTs, 12 explicitly mentioned the use of random	Reported on Page No.7

Section and Topic	Item #	Checklist item	Location where item is reported
		number tables to achieve randomization and were, therefore, judged as low-risk. The remaining 11 studies were classified as having ambiguous risk. In terms of allocation concealment, all studies were unclear because of a lack of detailed information. For the blind method of participants and staff and the blind method of result evaluation, the risks in all studies were unclear owing to a lack of details. For incomplete outcome data, two studies missed the follow-up time for recurrence rate, and the authors were not contacted. Thus, they were judged to be at a high risk. Finally, 21 studies reported complete outcome data, which were assessed as low risk. Finally, all studies on selective outcome reporting and other sources of bias were considered to be at risk of ambiguity owing to a lack of specific information. A summary of the risk of bias is shown in Figure 2.	
	20b	1.ORR<br>The ORR is a common indicator for evaluating tumor curative efficacy. 23 studies reported ORR. The ORR in the CT group was higher than that in the chemotherapy group (RR, 1.39;95%CI:1.28,1.52;p<0.00001,I2=3%). The heterogeneity between the studies was low, and the difference between the two groups was statistically significant. A forest plot of ORR is shown in Figure 3.<br>2.Recurrence rate and metastasis rate<br>Among the 23 included studies, three reported the recurrence rate, and the summary analysis implied that the recurrence rate of the CT group was lower than that of the control group(RR:0.33;95%CI:0.14,0.78;p=0.01, I2 = 0%). Four studies reported the tumor metastasis rate. Similarly, the metastasis rate of CT was lower than that of chemotherapy (RR, 0.48;95%CI:0.31,0.73;p=0.0006, I2 = 0%). There was no heterogeneity among the studies, and the combined data showed that the difference between the two groups was statistically significant. Forest plots of metastasis and recurrence rates are shown in Figure 4.<br>3.Average PFS<br>Six studies reported average PFS. Compared with the control group, a statistically significant increase in average PFS was observed in the CT group (SMD:2.78;95%CI:1.41,4.14; p < 0.0001, I2=97%). This result implies that additional TCM treatment to strengthen resistance and eliminate pathogenic factors may be beneficial for increasing the average PFS of patients with TNBC. However, a high degree of heterogeneity was observed among these studies. Moreover, after subgroup analysis according to disease stage or course, heterogeneity was still high. As no source of heterogeneity was found, this result should be treated with caution. A forest plot of the average PFS is shown in Figure 5.<br>4.KPS improvement<br>Five studies reported improvements in KPS scores. The meta-analysis indicated that the KPS score improved more in the CT group than in the control group, with a statistically significant difference between the two groups (RR:1.55;95%CI:1.21,1.99;p=0.0005, I2 = 52%). Moderate heterogeneity was observed among studies. A forest plot of KPS improvement is shown in Figure 6.<br>5..Tumor marker level<br>Level of CEA, CA15-3 and CA125<br>Six studies reported levels of CEA and CA15-3, and four studies reported the levels of CA125.The results indicated that the treatment group of CEA(SMD:-1.99;95%CI:-2.95,-1.03;p<0.0001,I2=96%),CA15-3(SMD:-1.52;95%CI:-2.31,-0.73; p=0.0002, I2=94%), and CA125(SMD: -1.36;95%CI:-2.18,-0.54; p < 0.001, I2＝92%) were all lower than those in the control group, and there were significant differences between the two groups. There was high heterogeneity among studies; after excluding studies of Wu et al. (2018) and Zhao (2021), the heterogeneity of CEA(SMD: -0.89;95%CI:-1.31,-0.47; p< 0.0001, I2=76%) and CA15-3(SMD: -0.85;95%CI:-1.21,-0.49; p＜0.00001, I2＝67%) levels decreased, which implied that they may be the sources of heterogeneity. Similarly, the heterogeneity of CA125(SMD: -0.86;95%CI:-1.28,-0.44; p < 0.0001, I2 = 68%) decreased after excluding the Zhao 2021 study. Our results suggest that CT can effectively reduce tumor marker levels. Forest plots of tumor marker levels are summarized in Figure 7.<br>6.Changes of T lymphocyte subsets<br>Serum CD3+, CD4+, CD8+ and CD4+/CD8+ ratio<br>Five studies investigated the percentage of CD3 + T cells in the peripheral blood. The meta-analysis showed no significant difference in the percentage of CD3+ cells between the treatment and control groups (SMD:2.37;95% CI:-0.08,4.81; p=0.06, I2 = 99%). Four studies reported on	Reported on Page No.8 to No.11

PRISMA 2020 Checklist

Section and Topic	Item #	Checklist item	Location where item is reported
		the percentage of CD4 + cells. The percentage of CD4 + cells was significantly higher in the treatment group than in the control group(SMD:2.20;95%credibility interval:0.90,3.50; p=0.0009,I2 = 94%). Five studies documented a change in the CD8 + percentage. The meta-analysis suggested a lower CD8 + percentage in the CT group than in the chemotherapy group(SMD: -3.12;95%credibility interval:-4.68,-1.55; p<0.0001, I2＝97). Finally, four studies reported changes in the CD4 + / CD8 + ratio. The results showed that the ratio of CD4+/CD8+ cells in the CT group was higher than that in the chemotherapy group(SMD:2.21;95%CI:0.33,4.08; p=0.02, I2 = 97). Overall, the percentage of serum CD4 + and the CD4 + / CD8 + ratio was higher than that of the control group, which was lower in the CD8 + percentage than in the control group, whereas the percentage of CD3 + cells was not significantly different between the two groups. Our results imply that additional TCM treatment may reduce the negative impact of chemotherapy on the immune system, thus playing a protective role on the immune system. However, despite data analysis using a random-effects model, SMD model, and subgroup analysis according to disease stage and course of each group, no source of heterogeneity was found. The high heterogeneity among the studies indicates that there are significant differences between them; therefore, we should be cautious about the significance of the results. Forest plots of the changes in T lymphocyte subsets are shown in Figure 8.<br><br>7.Toxic and side effects<br><br>To evaluate the protective effect of a traditional Chinese medicine formula for strengthening the body and eliminating pathogenic factors by using the incidence of toxicity and side effects. The incidence of toxicity and side effects was calculated as the number of patients experiencing toxic reactions divided by the total number of patients. The main toxic effects reported in the included studies were adverse digestive tract effects, bone marrow suppression, alopecia, leukocyte decline, and platelet reduction.<br><br>7.1.Gastrointestinal adverse reactions<br><br>Seven studies have reported the overall incidence of gastrointestinal adverse effects, eight studies recorded the incidence of mild to moderate (grade I, Ⅱ)gastrointestinal adverse reactions, nine studies recorded the incidence of moderate to severe (gradeⅢ,Ⅳ) digestive tract adverse reactions, The results of the meta-analysis suggested in the overall incidence of(RR:0.59;95%CI:0.47,0.73; P< 0.00001, I2 = 23%)with moderate to severe (grade Ⅱ,Ⅲ,Ⅳ) digestive tract adverse reactions incidence aspect, the incidence of gastrointestinal adverse reactions in the treatment group was lower than that in the control group, and the difference between the two groups was statistically significant. There was no statistically significant difference between the two groups in the incidence of mild (grade I) gastrointestinal adverse reactions between the two groups.<br><br>7.2.Myelosuppression<br><br>Three studies reported the total incidence of myelosuppression, six studies recorded the incidence of mild to moderate (grade I and II) myelosuppression, six studies recorded moderate to severe (grade III and IV) myelosuppression. The results showed that in terms of the total incidence (RR:0.61; 95%CI:0.43,0.85; P<0.00001, I2 = 6%) and the incidence of moderate and severe (grade III,IV) myelosuppression, the incidence of myelosuppression in the treatment group was lower than that in the control group, and the difference between the two groups was statistically significant. However, no significant difference was observed in the incidence of mild-to-moderate myelosuppression (grades I and II) between the two groups.<br><br>7.3.Other toxic and side effects<br><br>Finally, four studies reported the incidence of alopecia and leukocytosis, and three studies documented the incidence of thrombocytopenia. There was no significant difference in the incidence of hair loss or leukopenia between the treatment and control groups. The incidence of thrombocytopenia in the CT group was lower than that in the chemotherapy group and the difference between the two groups was statistically significant (RR,0.56;95%CI:0.32,0.96; P=0.03, I2 = 0%).<br><br>Overall, compared with chemotherapy, CT had lower toxicity and side effects in terms of gastrointestinal adverse reactions, myelosuppression, moderate and severe gastrointestinal adverse reactions, myelosuppression, and thrombocytopenia, whereas there was no significant difference in the incidence of mild gastrointestinal adverse reactions, myelosuppression, alopecia, and leukopenia between the two groups. Therefore, TCM may reduce the toxic side effects associated with chemotherapy. Forest plots of toxicity and side effects are shown in Figure 9.	
	20c	1.Average PFS<br><br>Six studies reported average PFS. Compared with the control group, a statistically significant increase in average PFS was observed in the CT	Reported on Page

Section and Topic	Item #	Checklist item	Location where item is reported
		group (SMD:2.78;95%CI:1.41,4.14; p < 0.0001, I2=97%). This result implies that additional TCM treatment to strengthen resistance and eliminate pathogenic factors may be beneficial for increasing the average PFS of patients with TNBC. However, a high degree of heterogeneity was observed among these studies. Moreover, after subgroup analysis according to disease stage or course, heterogeneity was still high. As no source of heterogeneity was found, this result should be treated with caution. A forest plot of the average PFS is shown in Figure 5.<br>2.Tumor marker level<br>Level of CEA, CA15-3 and CA125<br>Six studies reported levels of CEA and CA15-3, and four studies reported the levels of CA125.The results indicated that the treatment group of CEA(SMD:-1.99;95%CI:-2.95,-1.03;p<0.0001,I2=96%),CA15-3(SMD:-1.52;95%CI:-2.31,-0.73; p=0.0002, I2=94%), and CA125(SMD: -1.36;95%CI:-2.18,-0.54; p < 0.001, I2＝92%) were all lower than those in the control group, and there were significant differences between the two groups. There was high heterogeneity among studies; after excluding studies of Wu et al. (2018) and Zhao (2021), the heterogeneity of CEA(SMD: -0.89;95%CI:-1.31,-0.47; p< 0.0001, I2=76%) and CA15-3(SMD: -0.85;95%CI:-1.21,-0.49; p＜0.00001, I2＝67%) levels decreased, which implied that they may be the sources of heterogeneity. Similarly, the heterogeneity of CA125(SMD: -0.86;95%CI:-1.28,-0.44; p < 0.0001, I2 = 68%) decreased after excluding the Zhao 2021 study. Our results suggest that CT can effectively reduce tumor marker levels. Forest plots of tumor marker levels are summarized in Figure 7.<br>3.Changes of T lymphocyte subsets<br>Serum CD3+, CD4+, CD8+ and CD4+/CD8+ ratio<br>Five studies investigated the percentage of CD3 + T cells in the peripheral blood. The meta-analysis showed no significant difference in the percentage of CD3+ cells between the treatment and control groups (SMD:2.37;95% CI:-0.08,4.81; p=0.06, I2 = 99%). Four studies reported on the percentage of CD4 + cells. The percentage of CD4 + cells was significantly higher in the treatment group than in the control group(SMD:2.20;95%credibility interval:0.90,3.50; p=0.0009,I2 = 94%). Five studies documented a change in the CD8 + percentage. The meta-analysis suggested a lower CD8 + percentage in the CT group than in the chemotherapy group(SMD: -3.12;95%credibility interval:-4.68,-1.55; p<0.0001, I2＝97). Finally, four studies reported changes in the CD4 + / CD8 + ratio. The results showed that the ratio of CD4+/CD8+ cells in the CT group was higher than that in the chemotherapy group(SMD:2.21;95%CI:0.33,4.08; p=0.02, I2 = 97). Overall, the percentage of serum CD4 + and the CD4 + / CD8 + ratio was higher than that of the control group, which was lower in the CD8 + percentage than in the control group, whereas the percentage of CD3 + cells was not significantly different between the two groups. Our results imply that additional TCM treatment may reduce the negative impact of chemotherapy on the immune system, thus playing a protective role on the immune system. However, despite data analysis using a random-effects model, SMD model, and subgroup analysis according to disease stage and course of each group, no source of heterogeneity was found. The high heterogeneity among the studies indicates that there are significant differences between them; therefore, we should be cautious about the significance of the results. Forest plots of the changes in T lymphocyte subsets are shown in Figure 8.	No.8 to No.11
	20d	This content is included in the 20b section	Reported on Page No.8 to No.11
Reporting biases	21	This content is included in the 20b section	Reported on Page No.8 to No.11
Certainty of evidence	22	This content is included in the 20b section	Reported on Page No.8 to No.11

PRISMA 2020 Checklist

Section and Topic	Item #	Checklist item	Location where item is reported
**DISCUSSION**			
Discussion	23a	In China, TCM has been widely used as adjuvant therapy for chemotherapy in the treatment of TNBC. In addition, epidemiological investigations have shown that " body resistance weakened while pathogenic factors prevailing" is the most common TCM syndrome in patients with TNBC. Recent studies have revealed possible anti-tumor mechanisms of CMFs for strengthening vital energy and expelling pathogenic factors. Furthermore, researchers have found that drugs that strengthen vital energy and eliminate pathogenic factors may play an anti-tumor role in different ways.<br>1. Anti-tumor mechanisms of Righting drugs<br>Righting drugs can enhance the healthy qi of the body, prevent invasion by external pathogens, and protect the body. Their anti-tumor efficacy is mainly reflected in two aspects: (1)To regulate immune function and then resist the invasion of tumor cells by improving the levels of T and B lymphocytes, NB cells, and macrophages and regulating the levels of cancer suppression-related cytokines, such as IL-2, IL-12, and TNF-α. Song et al. showed that icariin from Epimedium, a yangtonic drug, can effectively regulate the tumor immunosuppression microenvironment. Its mechanism is related to the downregulation of PD-L1 expression, increasing the proportion of infiltrating CD4+ / CD8+ T cells and reducing the content of MDSC in tumors. (2) To reduce the toxicity and side effects of chemotherapy drugs in the body, organ-protective, and anti-fatigue effects . Chen et al. observed the effect of Ganoderma lucidum spore oil on the level of peripheral blood cells in S180 tumor-bearing mice after 5-FU chemotherapy and found that Ganoderma lucidum spore oil can reduce the toxic and side effects of leukopenia and thrombocytopenia caused by 5-FU chemotherapy, suggesting that righting drugs can improve the hematopoietic dysfunction of bone marrow caused by chemotherapy to a certain extent. Additionally, ganoderma acid reduced peripheral muscle fatigue-like behavior induced by 5-FU and improved mitochondrial function of the muscle by suppressing p-AMPK, IL-6, and TNF-α expression in the skeletal muscle, increasing glycogen content and ATP production, and reducing lactic acid content and LDH activity .<br>2.Anti-tumor mechanism of anti-pathogenic drugs<br>The anti-tumor effect of anti-pathogenic drugs is mainly reflected in the effective suppression of tumor cell self-proliferation and angiogenesis, induction of tumor cell apoptosis, and autophagy . A commonly used qi-tonifying drug for TNBC, Szechwan Chinaberry fruit, whose extracts have inhibitory effects on TNBC cells, such as MDA-MB-231, BT549, and 4T1 cells, can induce necrosis, apoptosis, and autophagy .<br>Moreover, it was found that both righting and anti-pathogenic drugs can reduce the resistance of TNBC to chemotherapy drugs, thus improving the efficacy of chemotherapeutic drugs. In summary, the anti-tumor mechanism of TCM is mostly related to immunity regulation, organ protection, fatigue resistance, tumor inhibition, and sensitivity to chemotherapy drug amplification.	Reported on Page No.13
	23b	First, the included studies had poor overall quality. Only a few of the included trials explicitly mentioned the specific method of randomization, and hidden and blinded information was lacking in all studies, which could lead to the generation of bias. However, it should be noted that it is difficult to hide the process of drug allocation in studies using TCM decoctions as interventions. Second, several results showed significant heterogeneity. The source of heterogeneity was not clearly defined despite the use of subgroup analysis and exclusion criteria. This phenomenon may be attributed to the differences between the various prescriptions used in each study. Significant heterogeneity reduced the strength of the evidence, suggesting that the results of the analysis should be interpreted with caution. Finally, because the included studies were conducted in China, it was difficult to evaluate the efficacy and safety of TCM in different ethnic groups and regions. Given these limitations, we recommend conducting large-scale, multicenter, high-quality clinical trials worldwide.	Reported on Page No.15
	23c	Second, several results showed significant heterogeneity. The source of heterogeneity was not clearly defined despite the use of subgroup analysis and exclusion criteria. This phenomenon may be attributed to the differences between the various prescriptions used in each study. Significant heterogeneity reduced the strength of the evidence, suggesting that the results of the analysis should be interpreted with caution. Finally, because the included studies were conducted in China, it was difficult to evaluate the efficacy and safety of TCM in different ethnic groups and regions. Given these limitations, we recommend conducting large-scale, multicenter, high-quality clinical trials worldwide.	Reported on Page No.15
	23d	This study indicated that TCM is a potential adjuvant chemotherapy therapy for TNBC, which not only improves the ORR, reduces the incidence of toxicity and side effects, recurrence and metastasis rate, and the level of tumor markers, but also enhances cellular immune function, prolongs PFS, and improves the quality of life. Although these conclusions require further confirmation owing to the existing limitations, the current evidence provides hope for clinical researchers to explore further.	Reported on Page No.16

PRISMA 2020 Checklist

Section and Topic	Item #	Checklist item	Location where item is reported
**OTHER INFORMATION**			
Registration and protocol	24a	This agreement was registered with PROSPERO under registration no. CRD42022327655.	Reported on Page No.3
	24b	PROSPERO	Reported on Page No.3
	24c	none	
Support	25	This study was supported by the National Natural Science Foundation of China (grant no. 22274013). This study was supported by the National Major Scientific and Technological Infrastructure for Translational Medicine (Shanghai) (grant no. tmsk-2021-411).	Reported on Page No.17
Competing interests	26	The authors declare no conflicts of interest regarding the publication of this paper.	Reported on Page No.16
Availability of data, code and other materials	27	The research data supporting this publication are available from CNKI, Wanfang Database, and CQVIP.<br>template data collection forms; search process are availability.	Reported on Page No.16

From: Page MJ, McKenzie JE, Bossuyt PM, Boutron I, Hoffmann TC, Mulrow CD, et al. The PRISMA 2020 statement: an updated guideline for reporting systematic reviews. BMJ 2021;372:n71. doi: 10.1136/bmj.n71

For more information, visit: http://www.prisma-statement.org/
