# Supplementary material for: Combination of Chinese medicinal formulas and chemotherapy for triple-negative breast cancer strengthens body resistance to eliminate pathogenic factors
Source: Medicine (Baltimore). 2022 Dec 23;101(51):e32350. doi: 10.1097/MD.0000000000032350 (PMC9794332; doi:10.1097/MD.0000000000032350)
Supplement: Supplementary file 2 [file medi-101-e32350-s002.pdf]

PRISMA 2020 flow diagram for new systematic reviews which included searches of databases and registers only

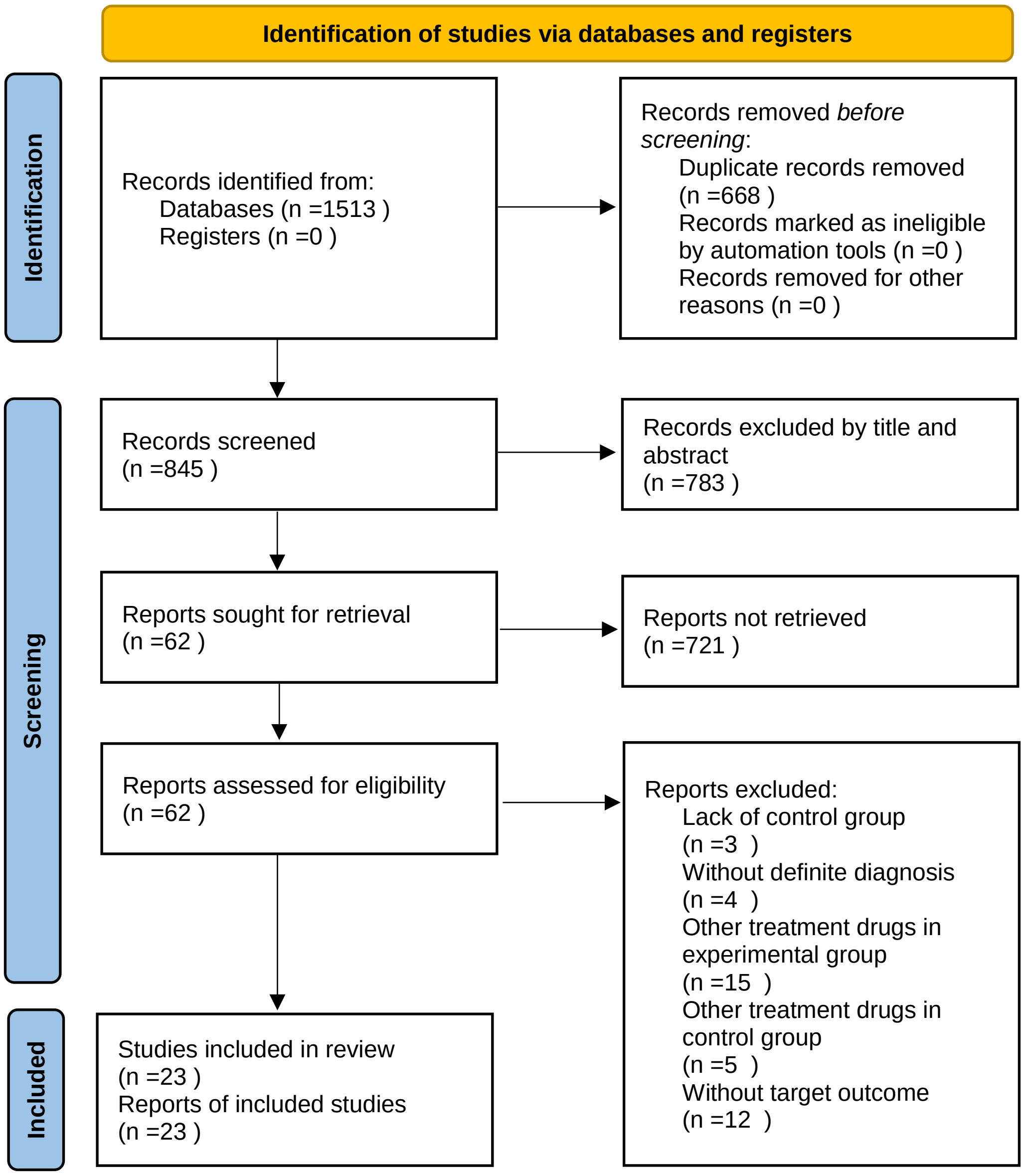

*Consider, if feasible to do so, reporting the number of records identified from each database or register searched (rather than the total number across all databases/registers).

**If automation tools were used, indicate how many records were excluded by a human and how many were excluded by automation tools.

From: Page MJ, McKenzie JE, Bossuyt PM, Boutron I, Hoffmann TC, Mulrow CD, et al. The PRISMA 2020 statement: an updated guideline for reporting systematic reviews. BMJ 2021;372:n71. doi: 10.1136/bmj.n71

For more information, visit: http://www.prisma-statement.org/
